# Tissue Sodium Content and Arterial Hypertension in Obese Adolescents

**DOI:** 10.3390/jcm8122036

**Published:** 2019-11-21

**Authors:** Sophie Roth, Lajos Markó, Anna Birukov, Anja Hennemuth, Peter Kühnen, Alexander Jones, Niky Ghorbani, Peter Linz, Dominik N Müller, Susanna Wiegand, Felix Berger, Titus Kuehne, Marcus Kelm

**Affiliations:** 1 Institute for Computational and Imaging Science in Cardiovascular Medicine, Charité–Universitätsmedizin 13353 Berlin, Germany; sophie.roth@charite.de (S.R.); anja.hennemuth@charite.de (A.H.); niky.ghorbani@charite.de (N.G.); titus.kuehne@dhzb.de (T.K.); 2 Deutsches Herzzentrum Berlin, Department of Congenital Heart Disease, 13353 Berlin, Germany; berger@dhzb.de; 3 DZHK (German Centre for Cardiovascular Research), partner site Berlin, 10785 Berlin, Germany; lajosmarko@yahoo.com (L.M.); anna.birukov@charite.de (A.B.); dominik.mueller@mdc-berlin.de (D.N.M.); 4 Max Delbruck Center for Molecular Medicine, 13092 Berlin, Germany; 5 Berlin Institute of Health (BIH), 10178 Berlin, Germany; 6 Experimental and Clinical Research Center, a joint cooperation between the Charité Medical Faculty and the Max Delbruck Center for Molecular Medicine, 13125 Berlin, Germany; 7 Department of Paediatrics, Charité–Universitätsmedizin Berlin, 13353 Berlin, Germany; peter.kuehnen@charite.de (P.K.); susanna.wiegand@charite.de (S.W.); 8 Department of Paediatrics, University of Oxford, Oxford OX3 9DU, UK; alexander.jones@paediatrics.ox.ac.uk; 9 Institute of Radiology, Friedrich-Alexander-University Erlangen-Nürnberg, 91054 Erlangen, Germany; Peter.Linz@uk-erlangen.de

**Keywords:** obesity, sodium, hypertension, adolescents, MRI, MR-spectroscopy

## Abstract

Early-onset obesity is known to culminate in type 2 diabetes, arterial hypertension and subsequent cardiovascular disease. The role of sodium (Na^+^) homeostasis in this process is incompletely understood, yet correlations between Na^+^ accumulation and hypertension have been observed in adults. We aimed to investigate these associations in adolescents. A cohort of 32 adolescents (13–17 years), comprising 20 obese patients, of whom 11 were hypertensive, as well as 12 age-matched controls, underwent ^23^Na-MRI of the left lower leg with a standard clinical 3T scanner. Median triceps surae muscle Na^+^ content in hypertensive obese (11.95 mmol/L [interquartile range 11.62–13.66]) was significantly lower than in normotensive obese (13.63 mmol/L [12.97–17.64]; *p* = 0.043) or controls (15.37 mmol/L [14.12–16.08]; *p* = 0.012). No significant differences were found between normotensive obese and controls. Skin Na^+^ content in hypertensive obese (13.33 mmol/L [11.53–14.22] did not differ to normotensive obese (14.12 mmol/L [13.15–15.83]) or controls (11.48 mmol/L [10.48–12.80]), whereas normotensive obese had higher values compared to controls (*p* = 0.004). Arterial hypertension in obese adolescents is associated with low muscle Na^+^ content. These findings suggest an early dysregulation of Na^+^ homeostasis in cardiometabolic disease. Further research is needed to determine whether this association is causal and how it evolves in the transition to adulthood.

## 1. Introduction

Since the 1980s, the prevalence of obesity in many countries has doubled, affecting an estimated 603.7 million adults and 107.7 million children [1]. Globally, four million deaths per year have been attributed to increased body mass index (BMI), often due to associated cardiovascular disease (CVD) [1]. It is assumed that the course for the development of CVD is already set in childhood, but the exact mechanisms have remained unknown [1]. Amongst others, there are indications for chronic over-activity of the sympathetic nervous system and the renin–angiotensin–aldosterone system (RAAS) [2], as well as obesity-driven low-grade systemic inflammation-promoting type 2 diabetes [3].

Nutrition is known to play an important role in the pathogenesis of obesity, and mineral sodium (Na^+^) has been discussed as an essential risk factor for CVD. Many factors may mediate the ability of high salt intake to increase blood pressure, however, their relative contributions to the pathogenesis of salt-induced hypertension are controversial [4,5]. Although several studies report deleterious effects, including a rise in blood pressure and the emergence of chronic kidney disease, some other studies have found more neutral effects [4]. Nevertheless, the rate of deaths from cardiovascular causes has been attributed to sodium consumption, and was found to be lower in regions and cultures with reduced salt intake [5]. In overweight adults, sodium-induced increases in circulating volume and hypertension have been described, which can elevate mortality by promoting left ventricular hypertrophy, altering vascular resistance and ultimately leading to heart failure [6].

Intake and accrual of Na^+^ over the life course may, therefore, be an important determinant of CVD risk. Measurement of Na^+^ status is routinely done by analysis of spot or 24 h urines. However, doubts have been raised about the suitability of 24 h urinary Na^+^ excretion for estimating exact salt intake [7]. Whether urinary Na^+^ excretion is at all reflecting tissue Na^+^ content is currently unknown.

In recent studies, ^23^Na-magnetic resonance imaging (MRI) has been introduced as a reliable, non-invasive method to quantify Na^+^ tissue content [8,9,10,11,12,13] that overcomes this limitation. It has been suggested that tissue Na^+^ accumulation with ageing could be implicated in the pathogenesis of refractory hypertension in adults [9]. However, these studies were of adult patients with advanced disease, where the independent effects of ageing, as well as hypertension and diet-related obesity, were difficult to separate. Only little is known about Na^+^ storage in children and adolescents. However, studies in younger populations would have the advantage of excluding the effects of advanced ageing or long-established disease. To overcome this knowledge gap, the study investigates the associations between Na^+^ storage, hypertension and obesity in adolescent patients.

## 2. Materials and Methods

In this cross-sectional study twenty, previously untreated obese (aged 13–17 [median 14] years) and twelve normal-weight “control” (aged 13–16 [median 15] years) adolescents were studied. Obese and control subjects were matched for sex and age. Obese patients were recruited prospectively from the social–paediatric centre (Charité–Universitätsmedizin Berlin) between 2014 and 2017, and they were further divided into two sub-groups, according to their blood pressure status. They were defined as obese if their body mass index (BMI) exceeded the 97th Kromeyer–Hauschild percentile according to sex and age [14]. Controls were patients undergoing routine clinical diagnostic MRI for non-cardiac and non-endocrinologic reasons that volunteered to have our ^23^Na-MRI protocol added. The primary outcome was the absolute muscle sodium content; the secondary outcome was skin sodium content. Subjects with implanted devices not compatible with MRI and those with claustrophobia were excluded. Written informed consent from participants or their legal guardians was obtained before enrolment. The study was approved by the institutional ethics review board of the Charité–Universitaetsmedizin Berlin (approval reference number EA2/036/14) and conducted according to the principles of the Declaration of Helsinki.

Additionally to known blood pressure status, at the time of MRI, the subjects were comfortably seated with back support for at least five minutes while arterial blood pressure was measured on the right upper arm using an oscillometric Dinamap pro-100 device (Critikon, Milwaukee, WI, USA). Cuff size was chosen appropriately to arm circumference. As there is still no single value threshold available in obese and overweight children (BMI >85th percentile) and even adult guideline values vary between ACC and ESC guidelines [14,15,16], in this study, hypertension was defined if systolic and/or diastolic values exceeded age-, gender- and height-specific (95th) [16] reference percentiles.

Anthropometric data were acquired using standardised clinical protocols and participants completed a questionnaire about salt intake, answering on a scale of 1–10 according to their agreement with the question asked. An oral glucose-tolerance test (OGTT) was performed and HbA1c, fasten insulin and glucose were measured. Homeostatic model assessment (HOMA) indices above 2.0 were considered hyperinsulinism.

All subjects rested for at least 15 min before their left calf was scanned at its widest circumference. Imaging was performed on a Philips Ingenia 3.0 Tesla MR scanner (Ingenia R 5.4, Philips Healthcare, Best, The Netherlands) with a ^23^Na send/receive knee-coil (Rapid Biomedical, Rimpar, Germany), following previously validated methods, using a 2D-spoiled gradient echo sequence (total acquisition time, TA = 20.5 min; echo time, TE = 2.138 ms; repetition time, TR = 100 ms; flip angle, FA = 90°; 196 averages, resolution: 3 × 3 × 30 mm^3^) [9,10]. Four calibration phantoms containing aqueous solutions of 10, 20, 30, and 40 mmol/L NaCl were scanned as reference standards, together with the subject’s calf. Simultaneously, tissue water content was measured by ^1^H-MRI, using a fat-saturated inversion-prepared SE sequence (inversion time, TI = 210 ms; TA = 6.27 min; TE = 12 ms; TR = 3000 ms; FA = 90°; 1 average, resolution: 1.5 × 1.5 × 5 mm^3^), as implemented by other investigators [11].

Using ImageJ (NIH, version 1.50i) and the anatomical image (T1-weighted spoiled gradient echo sequence) as guidance, regions of interest (ROI) were drawn as centrally as possible whilst excluding prominent vascular structures, which are rich in Na^+^. Relevant ROIs included the total leg, triceps surae muscle, as the largest muscle of the calf (with medial and lateral gastrocnemius and soleus; referred in the text as “muscle”), skin, tibial bone and subcutaneous fat. Muscle ROIs and subcutaneous ROIs were marked on the T1-weighted sequence, while skin ROI, total leg ROI and phantom ROIs were drawn on the Na^+^ image, as already described by other authors [11]. ROI areas were assessed through each ROI’s voxel count and measured in arbitrary units (AU).

The signal intensity of each ROI was measured and linear trend analysis was used to translate this intensity to a NaCl concentration, according to the calibration phantoms’ predefined contents of 10, 20, 30, and 40 mmol/L [17]. A calibration standard for tissue water was based on the water content of the 10 mmol/L NaCl tube. Previous studies have suggested ^1^H-MRI as a means to non-invasively assess tissue water content changes, based on a linear relationship between ^1^H-MRI measurements and actual water content [9]. Thus, the combination of water and Na^+^ measurements allows the differentiation of a water-dependent Na^+^ storage (e.g., oedema) from water-independent Na^+^ storage (e.g., bound to glycosaminoglycans) [18].

Since fat tissue is rather low in Na^+^ content, we assessed whether decreased Na^+^ content in muscle might be due to increased muscle fat accumulation. Muscle fat content was assessed by the ratio of fat-voxels and the total number of voxels within the muscle. As described by Kopp et al., ^1^H-T1-weighted tissue signal intensities greater than 30% above the intensity level of the phantom tubes were defined “fat-voxels” [19]. The ratio of fat-voxels and the total number of voxels within the muscle was used to assess fatty muscle degeneration.

Data are expressed as median and interquartile range (Q1–Q3) unless otherwise stated. Data were tested for normality using the Shapiro–Wilk and Shapiro–Francia tests. Data were analysed for stochastic dominance among the three groups by applying a non-parametric Kruskal–Wallis test, followed by a Bonferroni-corrected Dunn’s test as a nonparametric, pairwise, multiple comparison procedure. Pearson’s chi-square test was used with Fisher’s exact test for comparison of categorical variables. To plot the combined effects of BMI and hypertension on Na^+^ content in muscle, predictive margins were calculated and plotted. Correlations were assessed by non-parametric multivariate regression analysis. Multifactorial effects (and their 95% confidence interval, CI) were assessed using robust regression. Significance level was set at *p* = 0.05 and 95% confidence intervals were calculated. Inter-observer variability was evaluated for Na^+^ measurements of 25 different subjects using a Bland–Altman plot. Stata (Version 15.1, StataCorp, College Station, Texas, USA) was used for statistical analysis.

## 3. Results

**Figure 1:**
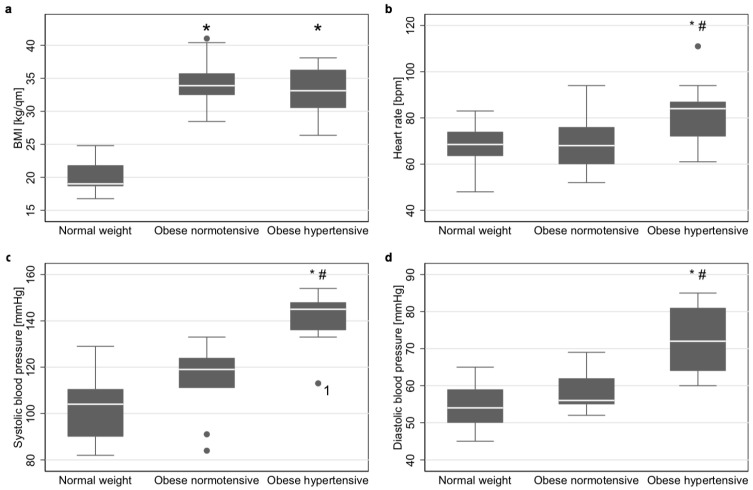
Group characteristics. (**a**) Boxplots of body-mass index (BMI) of controls, normotensive obese and hypertensive obese. (**b**) Boxplots of heart rate of controls, normotensive obese and hypertensive obese. (**c**) Boxplots of systolic blood pressure of controls, normotensive obese and hypertensive obese. (**d**) Boxplots of diastolic blood pressure of controls, normotensive obese and hypertensive obese. Tests were performed using a Kruskal–Wallis Test (N = 32) with inter-group p-values according to Bonferroni corrected Dunn’s test. * *p*-value < 0.05 compared to controls # *p*-value < 0.05 compared to normotensive obese. ^1^diastolic hypertension (above the 95th percentile).

Na^+^ content and tissue water content was analysed in a total of 32 subjects, of which 11 were hypertensive obese patients, nine normotensive obese patients and 12 normal-weight controls. The characteristics of these three groups are shown in Table 1. Age and sex did not significantly differ between groups, and BMI did not differ between both obesity groups (Figure 1a). Heart rate, systolic and diastolic blood pressure did not differ between normotensive obese and controls, but did between those two groups and hypertensive obese (Figure 1b–d, respectively). Only one patient had isolated diastolic blood pressure values exceeding the 95th percentile. All remaining hypertensive patients had systolic hypertension.

### 3.1. Tissue Na^+^, Water and Fat Content

We found significant differences in tibial bone Na^+^ content, subcutaneous fat Na^+^ content and water content of muscle, skin, tibial bone and subcutaneous fat in none of the groups (Supplementary Figure S1).

**Figure 2:**
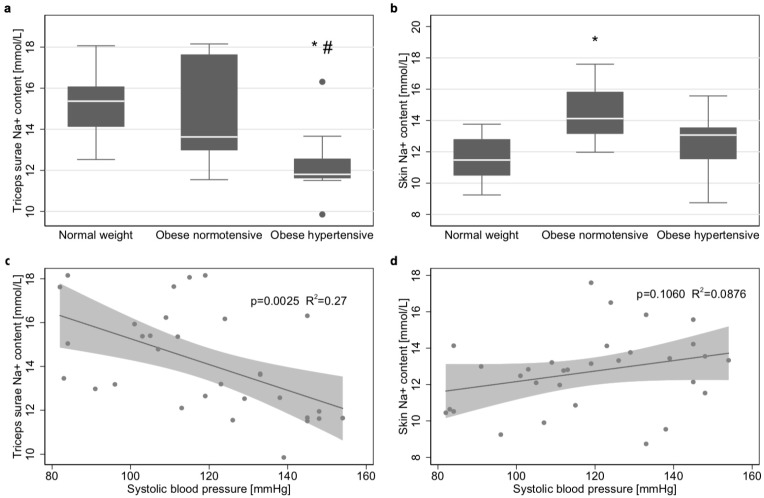
Na^+^ content in different tissues. (**a**) Boxplots of triceps surae muscle Na^+^ of controls, normotensive obese and hypertensive obese. (**b**) Boxplots of skin Na^+^ content of controls, normotensive obese and hypertensive obese. Tests were performed using a Kruskal–Wallis Test (N = 32) with inter-group *p*-values according to Bonferroni corrected Dunn’s test. * *p*-value <0.05 compared to controls # *p*-value <0.05 compared to normotensive obese. ^1^diastolic hypertension (above the 95th percentile). (**c**) The scatter plot and the linear regression model show an inverse correlation between triceps surae Na^+^ content and systolic blood pressure (*p* = 0.0025, R^2^ = 0.27, N = 32). (**d**) Scatter plot and the linear regression model, showing no significant correlation between skin Na^+^ content and systolic blood pressure (p = 0.1060 R^2^ = 0.09).

Muscle Na^+^ content did not differ (*p* = 1.00) between controls (15.37 mmol/L [interquartile range 14.12–16.08]) and normotensive obese (13.63 mmol/L [12.97–17.64]), but was significantly lower than both of these groups (*p* = 0.012 and *p* = 0.043, respectively) in hypertensive obese (11.95 mmol/L [11.62–13.66]) (Figure 2a). The Bland–Altman plot showed only low inter-observer variability in muscle Na^+^ content, due to variations in data processing (Supplementary Figure S2).

In contrast, skin Na^+^ content in normotensive obese (14.12 mmol/L [13.15–15.83]) was significantly higher (*p* = 0.004) than in controls (11.48 mmol/L [10.48–12.80]), while it tended to be higher in hypertensive obese (13.33 mmol/L [11.53–14.22]; *p* = 0.144). There was no difference between obese adolescents with or without hypertension (*p* = 0.237) (Figure 2b).

Cross-sectional total Na^+^ content of all compartments in hypertensive obese was 12.01 mmol/L [11.41–12.89] and was significantly lower than in normotensive obese (p = 0.045) and controls (*p* = 0.005). Total Na^+^ content did not significantly differ between controls (15.15 mmol/L [12.70–15.69]) and normotensive obese (13.02 mmol/L [12.39–14.98]; *p* = 0.866).

The proportion of fat tissue within the muscle did not vary between all three groups. No significant correlation was found (*p* = 0.149, R^2^ = 0.037) between the amount (area) of fat tissue and muscle sodium content (Table 1).

### 3.2. Na^+^ Content and Arterial Hypertension

As recent studies have reported correlations between Na^+^ accumulation and hypertension in adults [9], we further investigated these aspects in an adolescent cohort: an inverse correlation was found between muscle Na^+^ content and systolic blood pressure (*p* = 0.0025; R^2^ = 0.27; Figure 2c) and cross-sectional total sodium content (p=0.0173, R^2^=0.12). These effects were not attributed to BMI. In obese patients, no significant correlations were found between BMI and muscle Na^+^ content and total cross-sectional Na^+^ content. No significant correlation was found between skin Na^+^ content and systolic blood pressure (Figure 2d).

Since a relationship between arterial hypertension and heart rate increase was described in the literature for obesity, heart rates were also compared between groups2. The heart rate in hypertensive obese (84 [72–87]) was significantly higher (p = 0.008 and p = 0.025, respectively) than in controls (69 [64–74]) and normotensive obese (68 [60–76]). Between controls and normotensive obese, heart rate did not differ (*p =* 0.386, Figure 1b).

**Figure 3:**
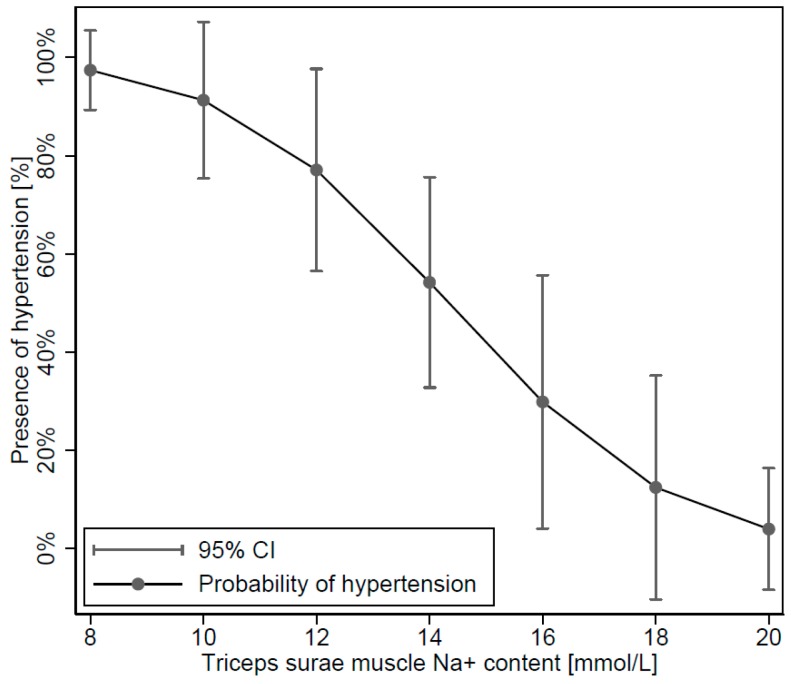
Logistic model showing the probability of hypertension according to muscle Na^+^ content with 95% confidence intervals (CI). (*p* = 0.038; N = 32).

A logistic regression model (Figure 3) in obese showed that the probability of hypertension was significantly associated with lower muscle Na^+^ content (*p* = 0.038). The risk for hypertension and its uncertainty is also shown in the figure, and was increased to >80% in patients with triceps surae muscle Na^+^ content below 12 mmol/L.

### 3.3. Sex Differences in Na^+^ Content and Arterial Hypertension

Sex differences were assessed for all storage compartments. No significant sex-specific differences were found for muscle Na^+^ content. Additionally, the associations between arterial hypertension and muscle sodium content, as well as total sodium content, were independent of the patient’s sex. Robust linear regression demonstrated associations (*p* < 0.001; R^2^ = 0.40) between skin Na^+^ content and independent variables of (1) female sex (Coef. −1.31 95% CI −2.52 to −0.10, *p* = 0.034) and (2) being normotensive obese (Coef. 2.69 95% CI 1.20 to 4.19, *p* = 0.001) but not (3) being hypertensive obese (*p* = 0.201). Tibial bone Na^+^ content was significantly lower in obese females (4.71 mmol/L [1.38–6.63]) than in obese males (7.45 mmol/L [6.10–9.14]; *p* = 0.025). Robust linear regression showed an association (*p* = 0.023; R^2^ = 0.25) between tibial bone Na^+^ content and independent variables of (1) female sex (Coef. −3.20, 95% CI −5.85 to −0.55, *p* = 0.020), but not (2) being hypertensive obese (*p* = 0.624) or (3) normotensive obese (*p* = 0.389). In other compartments, we did not find sex-specific differences in Na^+^ content.

### 3.4. Salt Intake, Tissue Na^+^ and Glucose Metabolism

The responses to the questionnaire items in all groups are shown in Table 1. In all items, no differences were found between groups regarding salt appetite or salty fast food craving.

**Figure 4:**
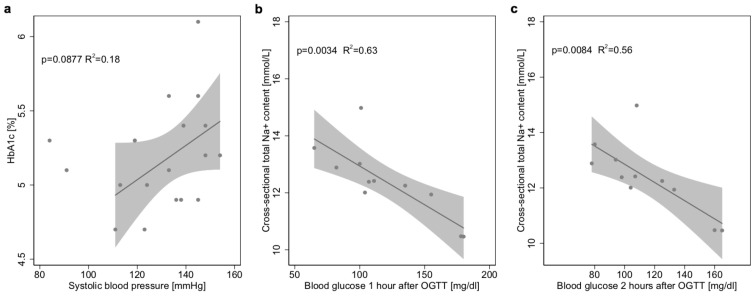
Tissue Na^+^ and glucose metabolism. (**a**) Linear regression model with 95% CI combined with scatter plot, showing no significant correlation between HbA1c and systolic blood pressure (n = 19). (**b**) Linear regression model with 95% CI, combined with scatter plot showing a significant correlation between Na^+^ of the whole leg and blood glucose 1 h after an oral glucose tolerance test (OGTT, *p* = 0.0034 R^2^ = 0.63, n = 11). (**c**) The linear regression model with 95% CI, combined with scatter plot showing a significant correlation between Na^+^ of the whole leg and blood glucose 2 h after an OGTT (*p* = 0.0084 R^2^ = 0.56, n = 11).

Hyperinsulinism, reflected by a HOMA index of 2.0 or higher, was found in 87% of all obese patients. We found a weak correlation between HbA1c and systolic blood pressure (*p* = 0.088; R^2^ = 0.18 n = 19; Figure 4a), but a stronger one between Na^+^ content of the whole leg and blood glucose levels after 1 h (*p* = 0.034; R^2^ = 0.63; n = 11; Figure 4b) and 2 h of OGTT (p = 0.084; R^2^ = 0.56; n = 11; Figure 4c). These effects were not found in other tissue compartments.

### 3.5. Power Calculation

A power calculation using a Satterthwaite’s t-test for unequal variances, based on relevant differences in muscle sodium content, and a group A (hypertensive obese) mean of 12.8 ± 1.877 and a group B (control) mean of 15.248 ± 1.657 mmol/L, a total sample size of N = 23 for both groups and a level of significance of 5% (two-sided), resulted in an estimated power of 92%. To estimate statistical power of differences between normotensive and hypertensive obese, a Group A (normotensive obese) mean of 14.9 ± 2.6190 and a Group B (control) mean of 15.248 ± 1.657 mmol/L, a total sample size of N = 20 for both groups and a level of significance of 5% (two-sided) resulted in an estimated power of 57%.

## 4. Discussion

Little progress has been made in preventing the emergence of cardiovascular risk factors, such as obesity and hypertension in their early stages [1,20]. The pathophysiological link between these conditions and the role of mineral Na^+^ intake remains incompletely understood, hampering the development of targeted prevention measures. In previous studies, tissue Na^+^ accumulation has been proposed as a factor in the pathogenesis of hypertension, based on findings of raised tissue Na^+^ in hypertensive adults [9], but its association with blood pressure status in childhood has not been studied before. We found that obese adolescents with hypertension had lower triceps surae muscle Na^+^ and lower total cross-sectional Na^+^ content than those without hypertension or normotensive controls. These effects were independent of the patients’ BMI. When comparing our results with those of hypertensive adults, one must be aware that these studies, to our knowledge, do not consistently provide information on patients’ weight [9]. Future studies should, therefore, further investigate to what extent tissue sodium storage in hypertensive and normotensive adults is linked to mechanisms of ageing and obesity.

### 4.1. Tissue Na^+^ Contents in Adults

^23^Na-MRI has been established as a non-invasive method for the quantification of Na^+^ content in different tissues [9,10,11,12,13,21]. Although our picture of Na^+^ homeostasis and tissue storage is still fragmented, this novel approach has made it possible to gain a considerable amount of knowledge in recent years. Tissue Na^+^ content was previously found to be elevated, among others, in patients with lipoedema [22], acute heart failure [11] or acute kidney disease [12] and chronic kidney disease [13]. In patients with refractory hypertension, women showed increased skin Na^+^ content compared to controls, whereas men showed higher muscle Na^+^ content compared to controls [9]. Increasing age further intensifies these differences in compartment distribution [17]. Additionally, sex differences have been described in a recent study [17]. In our study, we found higher skin and tibial bone Na^+^ content in males. Although these findings are in line with previous studies for the skin, the tibial bone has not yet been described as a compartment for sodium storage/depletion in patients with normal renal function, as its overall sodium content is typically much lower than that in the muscle/skin.

### 4.2. Na^+^ and Blood Pressure

The World Health Organization (WHO) recommends less than 2 g/day Na^+^ in adults, and even lower doses in children [23]. As the actual mean daily intake of Na^+^ is as high as 3.6 g/day in the US, UK, and Germany, we presume high salt intake also plays an important role in our study cohort [5]. Using questionnaires to semi-quantify the “salt appetite” of our participants, we did not observe any differences between the groups. However, to record the actual salt intake, complex measurements of nutritional composition would be required.

Salt-loading studies with salt-resistant subjects have shown that, during acute or chronic salt-loading, normotensive salt-resistant subjects do not excrete Na^+^ faster, nor do they experience more blood volume expansion. Instead, they substantially retain Na^+^ in a rhythmical manner, which is in line with our concept of relevant tissue Na^+^ buffers [24,25]. Impairment of these buffer mechanisms in our “early disease stage” study could be an important contributor to the onset of arterial hypertension: while, in obese young patients without hypertension, Na^+^ storage in the muscle (the largest previously described buffer compartment) is only slightly reduced, in those with hypertension we observed an accentuated reduction of Na^+^ storage in muscle. In other compartments, such as the skin, tibial bone and subcutaneous fat, Na^+^ did not show significant differences in hypertensive obese, excluding a potential Na^+^ shift to those compartments nearby.

Previous studies observed salt-sensitivity in some individuals, which describes an increase in blood pressure as a response to increases in dietary Na^+^ intake and vice versa [20,25,26]. In this context, the RAAS seems to be an important factor: it controls the vascular tone and—through Na^+^ reabsorption—the intravascular volume homeostasis, as well as the heart rate, through Angiotensin II activation [2,27]. Presuming similar Na^+^ intake in all groups, without a significant shift to other compartments, impaired muscle Na^+^ storage in hypertensive obese can result in elevated Na^+^ excretion through two major mechanisms: 1) altered RAAS suppression [28] and 2) pressure natriuresis as a regulatory response mechanism in salt-sensitive hypertensive subjects [29] through hypertension itself. Elevated heart rates in our cohort of hypertensive obese are in line with an overactivation of the sympathetic nervous system that has been described as an essential factor in the pathogenesis of obesity-induced hypertension in rabbits, dogs and humans [2].

In adults with long-term hypertension, recent reports suggest osmotically inactive Na^+^ storage in the skin via negatively charged glycosaminoglycans as binding partners [18]. We found elevated skin Na^+^ content in normotensive obese compared to normal-weight controls. However, these effects were not found in hypertensive obese. Therefore, no such conclusions can be made for skin Na^+^ content in adolescents.

Before Na^+^ transfers from the vascular system to tissue, there are two barriers to overcome: the negatively charged endothelial glycocalyx layer and the endothelial Na^+^ channel ENaC [30]. Therefore, Na^+^ homeostasis and salt sensitivity in arterial hypertension might be associated not only with a subnormal ability to excrete sodium load, but also with vascular endothelial dysfunction [24,27]. Whereas in patients with different stages of chronic kidney disease, salt restriction can reduce blood pressure [31], no controlled studies have been performed to demonstrate that hypertensive subjects excrete sodium more slowly and retain more of it than normotensive subjects.

One could, therefore, speculate whether regulatory mechanisms in adolescents are different from those observed in older patients. If the muscle, as a main Na^+^ storage compartment, stores less, maintenance of blood sodium homeostasis will depend more on excretion and other storage compartments. This may reflect a compensatory mechanism at an early stage of the disease-preventing tissue Na^+^ overload, and further research will be needed to investigate the hormonal cascades involved, as well as absolute Na^+^ excretion, as plasma Na^+^ needs to maintain stable.

### 4.3. Na^+^ Homeostasis and Hyperinsulinism

Insulin alone has long been thought to cause Na^+^ retention, with enough effects to contribute to arterial hypertension [32,33]. A study by Brands and colleagues showed that such Na^+^-retaining effects may be limited to uncontrolled Type II diabetes [32]. In 11 maintenance haemodialysis patients, higher muscle sodium content was, in a similar way, associated with insulin resistance [34]. Furthermore, in the soleus muscle of salt-sensitive hypertensive Dahl rats, the RAAS was shown to activate the NF-κB pro-inflammatory pathway, inducing moderate hyperinsulinemia and insulin resistance [33]. A recent study also demonstrated a positive statistical correlation between Na^+^ intake and insulin resistance in obese children and adolescents [35]. A majority (87.5%) of our disease cohort not only had hyperinsulinism, but were also showing a trend between HbA1c and blood pressure, even at this early stage of the disease.

Although the relation between obesity and hyper-caloric diets is known, the association between obesity and Na^+^ still remains unclear, as the quantification of salt intake is measured highly subjectively, through questionnaires or food records, unless investigators have the unusual opportunity of controlling their subjects’ nutrition completely, as done by Titze et al. in their space flight simulations [25]. In our study, we used salt questionnaires to assess the habits of salt intake and did not note any elevation of salt consumption or salty fast food craving in hypertensive or normotensive obese compared to our controls.

### 4.4. ^23^Na-MRI

Due to its non-invasive nature, ^23^Na-MRI is increasingly used in clinical research projects, even though it requires the use of specific non-proton coils and yields a certain complexity in image acquisition and post-processing [8,9,10,11,12,13,21].

Our study was performed at a 3 Tesla clinical scanner with a previously described measurement set-up [9,10]. A calibration curve using aqueous reference saline solutions was generated alongside to all scans, and 196 signal averages were applied to improve image homogeneity. We also determined intra- and inter-observer variability that did not show any significant differences between measurements. Compartments were assessed separately, as the total-cross-sectional Na^+^ content (shown for completeness) can be prone to averaging artefacts when different tissue types are combined, and the effects of the largest storage compartments (the muscle) may dominate.

### 4.5. Limitations

Within this study, we did not perform interventions such as salt loading tests, and, furthermore, assessments of the RAAS and Na^+^ excretion were not part of the study protocol. As indicated by our findings, future prospective and longitudinal studies should further evaluate the impact of such interventions and assess the complex interplay of hormonal axes (including the RAAS), as well as their linkage to the inflammation and glucose/insulin metabolism, as this may be of high relevance in future treatment-planning strategies. Furthermore, and as already stated above, salt intake was not measured quantitatively.

Statistical power to assess differences in muscle sodium content between controls and hypertensive obese was 92%, however, it was 57% for differences between controls and normotensive obese, due to sample size and the large heterogeneity within normotensive obese. These findings may be of use for future study planning.

## 5. Clinical Outlook and Conclusions

In obese adolescents with hyperinsulinism, arterial hypertension occurs in the presence of low muscle tissue Na^+^ content, independent of the patient’s BMI. These findings suggest a different regulation of muscle Na^+^ homeostasis and storage in early-onset obesity for hypertension compared to normotensive patients, adding a new perspective to salt-induced hypertension and salt sensitivity, if main storage compartments store less. The role of Na^+^ storage, salt sensitivity and insulin resistance in the initial stages of arterial hypertension, and the extent of the compensatory mechanisms that can become maladaptive at a later stage of the disease, as well as differences to isolated arterial hypertension in adults, remain to be further investigated in future clinical research.

## Figures and Tables

**Table 1:** Baseline characteristics. Median (interquartile range), unless stated otherwise.

Subjects	Controls (n = 12)	Normoten–Sive Obese (n = 9)	Hyperten–Sive Obese (n = 11)	*p* Value Normotensive Obese vs. Controls	*p* Value Hypertensive Obese vs. Controls	*p* Value Hypertensive Obese vs. Normotensive Obese	*p* Value Overall
Age [years]	15 (14–16)	15 (14–16)	14 (13–14)	1.000	0.088	0.060	0.072
Male Gender (n)	4 (33%)	4 (44%)	5 (45%)				0.808
Cross-Sectional Total Leg Area (mm^2^)	3830 (3536–4387)	6862 (5917–7244)	5912 (5217–6369)	<0.001	0.0007	0.533	<0.001
Tibial Bone Area (mm^2^)	229 (208–244)	311 (245–322)	284 (225–317)	0.011	0.117	0.478	0.022
Total muscle area (mm^2^)	2265 (1989–2482)	3196 (2780–3565)	2960 (2750–3377)	0.002	0.003	1.000	0.001
Subcutaneous Fat Area (mm^2^)	1084 (950–1363)	2526 (2078–3787)	2128 (1824–2939)	<0.001	0.001	0.758	<0.001
**Questionnaire**							
“How much do you like salty food?”	7 (5.5–7.5)	6 (5–8)	5 (3–8)	1.000	0.400	0.352	0.419
“How often do you add more salt to your food”	3.5 (2–6.5)	3(1–5)	2 (1–3)	0.497	0.059	0.504	0.129
“How much do you like salty snacks such as crisps?”	8 (5.5–8)	5 (4–7)	7 (4–9)	0.242	0.916	0.552	0.379
“How often do you eat in fast food restaurants?”	4 (2.5–4.5)	4 (3–5)	3 (2–4)	1.000	0.088	0.126	0.124
“How much money do you usually spend there?” (€)	5 (2.8–8)	9 (5–10)	5 (4–9.5)	0.197	1.000	0.161	0.218
“How often do you drink beverages without additional flavour?”	7 (5–9.5)	10 (9–10)	10 (8–10)	0.166	0.058	1.000	0.122
“How much do you drink daily?” (L)	1.5 (1.1–2)	1.5 (1–2.5)	2 (1–2.3)	0.601	0.524	1.000	0.587

Questionnaire on a scale from 1–10 (1 = I strongly agree; 10 = I strongly disagree) unless stated otherwise.

## Supplementary Materials

The following are available online at https://www.mdpi.com/2077-0383/8/12/2036/s1, Figure S1: Na^+^ and water content in different tissues, Figure S2: Bland-Altman Plot illustrating the inter-observer variability in the post-processing of the ^23^Na MRI data, Figure S3: Sex-specific tibial bone Na^+^.
